# Protocol for a feasibility study of a self-help cognitive behavioural therapy resource for the reduction of dental anxiety in young people

**DOI:** 10.1186/s40814-016-0054-2

**Published:** 2016-03-01

**Authors:** Zoe Marshman, Annie Morgan, Jenny Porritt, Ekta Gupta, Sarah Baker, Cathy Creswell, Tim Newton, Katherine Stevens, Christopher Williams, Suneeta Prasad, Jennifer Kirby, Helen Rodd

**Affiliations:** 1 School of Clinical Dentistry, University of Sheffield, Claremont Crescent, Sheffield, S10 2TA UK; 2 Paediatric Dentistry Department, Charles Clifford Dental Hospital, Wellesley Road, Sheffield, S10 2SZ UK; 3 Department of Psychology, Sociology, and Politics, Sheffield Hallam University, Room 2.05 Heart of the Campus, Collegiate Crescent, Sheffield, S10 2BQ UK; 4 School of Psychology and Clinical Language Sciences, University of Reading, Earley Gate, Whiteknights, Reading, Berkshire, RG6 6AL UK; 5 Oral Health Services Research and Dental Public Health, King’s College London, Denmark Hill Campus, Caldecot Road, London, SE5 9RW UK; 6 School of Health and Related Research (ScHARR), University of Sheffield, Regent Court, 30 Regent Street, Sheffield, S1 4DA UK; 7 Institute of Health and Wellbeing, Mental Health and Wellbeing, University of Glasgow, Gartnavel Royal Hospital, Administration Building, 1055 Great Western Road, Glasgow, G12 0XH UK; 8 Derbyshire Community Health Services, Long Eaton Dental Clinic, Midland Street, Long Eaton, Nottingham, NG10 1RY UK

**Keywords:** Dental anxiety, CBT, Children, Young people, Feasibility study, CBT self-help, Low intensity, Qualitative research

## Abstract

**Background:**

Childhood dental anxiety is very common, with 10–20 % of children and young people reporting high levels of dental anxiety. It is distressing and has a negative impact on the quality of life of young people and their parents as well as being associated with poor oral health. Affected individuals may develop a lifelong reliance on general anaesthetic or sedation for necessary dental treatment thus requiring the support of specialist dental services. Children and young people with dental anxiety therefore require additional clinical time and can be costly to treat in the long term. The reduction of dental anxiety through the use of effective psychological techniques is, therefore, of high importance. However, there is a lack of high-quality research investigating the impact of cognitive behavioural therapy (CBT) approaches when applied to young people’s dental anxiety.

**Methods/design:**

The first part of the study will develop a profile of dentally anxious young people using a prospective questionnaire sent to a consecutive sample of 100 young people referred to the Paediatric Dentistry Department, Charles Clifford Dental Hospital, in Sheffield. The second part will involve interviewing a purposive sample of 15–20 dental team members on their perceptions of a CBT self-help resource for dental anxiety, their opinions on whether they might use such a resource with patients, and their willingness to recruit participants to a future randomised controlled trial (RCT) to evaluate the resource. The third part of the study will investigate the most appropriate outcome measures to include in a trial, the acceptability of the resource, and retention and completion rates of treatment with a sample of 60 dentally anxious young people using the CBT resource.

**Discussion:**

This study will provide information on the profile of dentally anxious young people who could potentially be helped by a guided self-help CBT resource. It will gain the perceptions of dental care team members of guided self-help CBT for dental anxiety in young people and their willingness to recruit participants to a trial. Acceptability of the resource to participants and retention and completion rates will also be investigated to inform a future RCT.

## Background

Dental anxiety is common, affects people of all ages [1], and tends to develop in childhood and adolescence [2, 3]. The prevalence of severe dental anxiety in children ranges from 5 to 20 % in different countries and age groups [4–6]. Dental anxiety impacts on the quality of life of young people with those affected reporting significantly worse oral health-related quality of life, with particular impact on social and emotional well-being such as feeling upset or worried or being teased or left out [7]. Young people with high levels of dental anxiety also have more decayed and extracted teeth [8, 9] and demonstrate increased unmet need for dental care [10, 11] as compared to their peers with low levels of anxiety and who are regular attenders. Up to one in five young people reports not visiting the dentist regularly because of fear [12]. Fear is also a significant barrier to completion of dental treatment [13]. Research has found that children who are most likely to have missed previous dental appointments were those who were rated by their parents as having high levels of dental anxiety [14]. Hallberg and colleagues [15] found that parents experienced difficulties trying to persuade their dentally anxious children to go to their dental appointment and felt they lacked strategies to handle their child’s level of fear and effectively manage the situation [16].

Dental anxiety in young people also has a significant impact on dental services. Providing treatment for anxious patients is time consuming, costly, demanding, and a cause of occupational stress for dentists [17]. These factors result in patients being referred to secondary care services, having to wait longer for dental treatment and increased costs to the National Health Service (NHS) [18].

Traditionally, dental anxiety has been managed using pharmacological techniques including inhalational sedation and general anaesthetic (GA). However, such approaches only manage rather than reduce children’s dental anxiety [19]. The cost per case for inhalational sedation or GA for dental treatment has been estimated at £273 and £720, respectively [20]. Indeed, dental anxiety remains unchanged in those children who receive dental treatment under GA [21] with anxious children becoming adults with a long-term reliance on expensive interventions in specialist settings.

Over recent years, it has been recognised that greater effort should be directed towards behaviour management and psychological interventions which can reduce the patient’s anxiety in the long term [22, 23]. Advances in the treatment of anxiety in young people have been made in the field of cognitive behavioural therapy (CBT), including self-help approaches. CBT is a goal-orientated talking therapy which aims to help people manage their problems by changing how they think and behave in relation to their problems. It is a type of therapy which can be used to teach patients (and often their parents/carers) skills for the self-management of their anxiety. CBT incorporates a variety of different cognitive and behavioural strategies which aim to help the patient modify the unhelpful behaviours or thoughts maintaining their anxiety [24].

A review of meta-analyses revealed that CBT is highly effective in treating a range of anxiety disorders in both children and adults [25, 26]. Behavioural interventions, such as graded exposure and systematic desensitisation to feared stimuli, have been found to be effective in reducing dental anxiety levels of children [27–29]. Studies have also found modelling interventions, which help patients develop effective coping skills by observing other people (e.g. other children, parents) successfully receiving dental treatment (film, in vivo), are effective in improving the behaviour of dentally anxious young people [30]. Whilst there has been a paucity of research investigating the effectiveness of cognitive-behavioural interventions in reducing young people’s dental anxiety, research has revealed promising results in the use of such strategies to reduce adults’ dental anxiety [31, 32].

Based on (1) evidence for the effectiveness of self-help CBT for young people with general anxiety; (2) case studies of CBT for children with dental anxiety [33, 34]; and (3) experience from the use of CBT for dental anxiety in adults including CBT delivered by dental nurses in Sheffield, there was sufficient evidence to suggest that a self-help CBT resource for young people with dental anxiety could be highly effective at reducing dental anxiety and its consequences. However, no such resource had been evaluated to date.

### Aims of study

The study will determine the feasibility of evaluating, in a randomised controlled trial (RCT), a CBT resource for young people (aged 9–16 years) for the reduction of dental anxiety.

It will specifically:

- Identify a profile of dentally anxious young people who could potentially be helped by a guided self-help CBT resource
- Explore perceptions of primary dental care team members of guided self-help CBT for dental anxiety in young people and their willingness to recruit participants to a future substantive trial
- Evaluate the retention and completion rates and acceptability of the guided self-help CBT resource to patients and parents
- Identify potential outcome measures for use in a future RCT
- Determine the sample size required for a future RCT

## Methods/design

This mixed-method study will be conducted in three phases to allow the objectives to be achieved and to capture the perspectives of all relevant stakeholders. For each phase the design, procedure and approach to data analysis will be described. Ethical approval will be obtained from an NHS research ethics committee.

### Intervention

The intervention will be a self-help paper-based CBT resource developed with young people, parents, and dental professionals based on the Five Areas model of CBT [24] which uses a combination of cognitive and behavioural techniques to reduce dental anxiety. The resource will be given to young people by a dentist who will guide them through the use of it during dental appointments. More information about the resource can be found at https://www.sheffield.ac.uk/dentalschool/research/create/research_projects.

A) ***The profile of dentally anxious young people***
**Design of the study**
    This part of the project will develop a profile of dentally anxious young people who could potentially be helped by a self-help CBT resource to reduce dental anxiety. It will include a prospective questionnaire sent to a consecutive sample of 100 young people referred to the Paediatric Dentistry Department in Sheffield for the management of dental anxiety. The questionnaire will include items to establish patient demographic information, quality of life (the Child Health Utility 9D) [35], level of dental anxiety (Modified Child Dental Anxiety Scale) [36], any psychological disorders (Revised Child Depression and Anxiety Scale and the Strengths and Difficulties Questionnaire) [37, 38], and willingness to consider using a CBT resource. The findings will allow an estimate to be made of the number of young people and their parents who could potentially be helped by a guided self-help CBT resource and that would need to be assessed in order to identify sufficient patients who are eligible and willing to take part in a future trial.
**Setting and recruitment**
    Suitable child patients will be identified by a clinician member of the study team by screening of referral letters to the Paediatric Dentistry Department according to the inclusion/exclusion criteria described below. The questionnaire will be posted to potential participants, with covering letters and Participant Information Sheets for both the young person and their parent/carer. Young people will be involved in the design of the covering letter and Participant Information Sheets. Parents/carers will be invited to help their child complete the questionnaire if necessary. If young people and their parent consent for them to take part, they will be asked to return the completed questionnaire when they attend for their appointment. In order to maximise the response rate, if the participants have not completed their questionnaire when they attend for their appointment, they will be given a copy to complete at the appointment and sufficient time to do this in a private area of the clinic. Based on previous surveys in the department, a response rate of 60 % is anticipated. Participants will continue to be recruited until the sample size of 100 is achieved. Recruitment will be restricted to those aged 11–16 years due to the length of the questionnaire which takes around 15 min to complete and the nature of some of the questions about depression and anxiety.
**Sample size**
    The sample size has been based on a similar questionnaire survey of 100 adults attending a clinic for patients with dental anxiety at a dental hospital in London [39].
**Type of participants**
    The following inclusion and exclusion criteria will be adopted:
*Inclusion criteria*
    Children aged 11–16 years old with mild to moderate dental anxiety
    Children referred to the Paediatric Dentistry Department by a primary or secondary dental care provider for the management of dental anxiety
*Exclusion criteria*
    Children who are unable to complete a questionnaire due to learning or language difficulties
**Data analysis**
    Data will be entered into an electronic database (Statistical Package for Social Sciences, v20) and analysed using simple descriptive statistics.

B) ***The perceptions of dental care team members***
**Design of the study**
    This part of the study involves a qualitative study of the perceptions of primary and secondary dental care team members of the developed self-help CBT resource and their willingness to recruit participants to a trial and potentially offer the resource to their own patients. This will be accomplished through conducting and analysing individual interviews.
    Face-to-face individual or group interviews will be conducted with members of the primary and secondary care dental care teams using a topic guide as the framework for the interview. A combination of individual and group interviews will be employed to gain a breadth and depth of information and at the convenience of the participants. The topic guide will be developed based on the theory of planned behaviour to explore the key barriers and facilitators (e.g. attitudes, perceived behavioural control, subjective norms) which may influence dental team members’ intentions to use a self-help CBT resource with their patients in future [40]. The interviews will be digitally recorded and transcribed verbatim as quickly as possible after the event and the recordings deleted after a short time. All identifying information will be removed from the transcripts to ensure anonymity.
**Setting and recruitment**
    The research participants will be recruited from general dental practices, the salaried dental service, and secondary dental care in South Yorkshire. Participants will be purposively recruited to include dental practices serving varying levels of deprivation and ethnic minorities. The goal of purposive sampling will be to provide a range of experiences and views. Sufficient participants should be involved to achieve saturation of information but not so many to prohibit detailed analysis. It is expected that 15–20 dental care professionals will be interviewed.
    1. Identification: practices will be identified from the list of providers in South Yorkshire.
    2. Approaching: potential participants will be sent written information in the post.
    3. Recruitment: after the initial approach, the dental care professional will then be contacted by telephone by a researcher, after being given at least 1 week to consider whether or not they want to take part, with the aim of arranging a date for the interview. Written consent will be obtained on the day of the interview. Interviews will be conducted by trained researchers with both dental and social science backgrounds to reduce the risk of influencing the results. Dental care professionals will be given thank-you vouchers in gratitude for their time.
**Sample size**
    As a qualitative approach is being used, no formal sample size calculation has been carried out. Based on previous experience, a maximum of 20 participants is likely to be required to achieve saturation [41, 42]. Recruitment will continue until no new themes emerge.
**Type of participants**
    The following inclusion and exclusion criteria will be adopted:
*Inclusion criteria*
    Dental professionals who are primarily employed in general dental practice or the salaried dental service in Sheffield
    Dental professionals who provide dental treatment to children and young people
    Dental professionals who have made a referral to a secondary dental care provider for the management of dental anxiety of a child or young person
*Exclusion criteria*
    Dental professionals who do not treat children aged 9–16 years
    Dental professionals who do not provide NHS treatment
**Data collection**
    Interviews will be conducted with individuals or in groups and arranged at a mutually convenient time and place, preferably at the participant’s place of work. The interview will be recorded using a digital sound recorder and will last 30–60 min.
**Data analysis**
    The data will be analysed using framework analysis [43]. The framework will be informed by the theory of planned behaviour which has been used previously to explore dental practitioner’s clinical practice [44]. Two researchers will be involved in the analysis of the qualitative data.

C), D), and E) ***Evaluation of retention rates and completion rates, acceptability of the guided self-help CBT resource, evaluation of potential outcome measures, and sample size calculation***
**Design of the study**
    This stage will comprise a pre-post test single group design and will involve asking a consecutive sample of young people and their parents to complete the resource to evaluate retention, completion rates, and acceptability, to evaluate potential outcome measures and provide data to allow a sample size calculation for an RCT. Participants will be asked to complete a battery of measures and will then be given the resource to work through with their parent or carer and clinician. After they have completed three visits for dental treatment, they will then complete the questionnaire a second time. The questionnaire will include patient demographic information, dental anxiety (Modified Child Dental Anxiety Scale [45]), and health-related quality of life (Child Health Utility 9D) [46]. A sample of young people and parents/carers will also be interviewed to explore the acceptability of the resource. The interviews will be digitally recorded and transcribed verbatim as quickly as possible after the event and the recordings deleted after a short time. All identifying information will be removed from the transcripts to ensure anonymity.
**Setting and recruitment**
    The research participants will be recruited purposively from new patients to a general dental practice in South Yorkshire, Derbyshire, salaried dental service and the Paediatric Dentistry Department of the Charles Clifford Dental Hospital in Sheffield. The sample will be recruited to involve young people living in areas with a range of different levels of deprivation and from different black and ethnic minority groups. It is expected that a total of 100 families will be recruited.
    1. Identification: potential participants will be identified by their dentist based on the inclusion and exclusion criteria.
    2. Initial approach: potential participants and their parents will be approached by a research nurse and the young person will be asked ‘Do you feel worried or afraid about going to the dentist?’ If they say ‘yes’ then they will be invited to take part in the study.
    3. Recruitment: after the initial approach, the children and their parents will be given separate written information sheets to take away and read.
    4. At the next dental appointment, which is part of the child’s normal course of planned treatment and will typically involve a non-anxiety-provoking preventive treatment, child and parental consents will be obtained from those wishing to participate in the study. At this time, the CBT resource will be provided and the participant will also complete the baseline questionnaire (T_1_).
    5. The participant will continue along their normal care pathway for their course of treatment (which could include treatment under inhalation sedation) and will work through the CBT resource at subsequent visits with the same dentist. At their third visit, they will complete the same questionnaire again (T_2_) and continue with their course of treatment as normal. The time interval between T_1_ and T_2_ will vary between participants.
**Sample size**
    For the qualitative aspect, no formal sample size calculation has been carried out although previous studies have required interviews with 10–15 participants before data saturation is reached [47, 48].
    In order to evaluate the potential outcome measures and determine the sample size for a future RCT, a total sample size of 60 participants will be sought. A sample size of 50 is recommended by Consensus-based Standards for the selection of health measurement instruments [49]. Allowing for a 40 % drop-out rate between baseline and follow-up, the intent is to recruit 100 participants.
**Type of participants**
    The following inclusion and exclusion criteria will be adopted:
*Inclusion criteria*
    Children aged 9–16 years old with mild to moderate dental anxiety
    Children requiring a course of dental treatment which requires at least three separate visits
*Exclusion criteria*
    Children diagnosed with an underlying psychological disorder
    Children with an acute dental problem who require urgent dental treatment
    Children with a severe disability where communication is not possible
    Non-English speaking children and parent/carers (to avoid the need for translating services where the participant’s responses might be unintentionally altered as a result of being translated)
**Data collection**
    Data will be collected about the following outcomes: dental anxiety, health-related quality of life, acceptability, missed or cancelled appointments, completion of the course of treatment, and the level of engagement with the resource.
    A battery of outcome measures including the Modified Child Dental Anxiety Scale [36] and the Child Health Utility 9D [46] will be given to the participants to complete then the CBT resource will be introduced along with the accompanying instructions for young people and their parents. Interviews will be arranged once the participants have completed their course of dental treatment and as much of the resource as they want. Data will also be recorded by a member of the clinical care team to include: missed or cancelled appointments, completion of the course of treatment, and the level of engagement with the resource.
    The interviews will take place at a mutually convenient time and place, preferably at the young person’s home. The interview will be recorded using a digital sound recorder and will last for 45–60 min. Interviews will be conducted with both parents and children.
**Data analysis**
    The qualitative data will be analysed using framework analysis [43]. Two researchers will be involved in the analysis of the qualitative data. Quantitative data will be entered into an electronic database (Statistical Package for Social Sciences, v20) and analysed using simple descriptive statistics, internal consistency (Cronbach’s alpha) and validity through correlation coefficients to evaluate the performance of the measures. Our main outcomes of interest are take-up, recruitment rates, use of the resource, completion rates, and our ability to gather data at baseline and follow-up.

## Discussion

This study protocol is designed to investigate the feasibility of evaluating, in an RCT, a CBT resource for young people (9–16 years) for the reduction of dental anxiety. The findings will provide valuable information regarding recruitment (including sample size), the most appropriate outcome measures, retention and completion rates, and the acceptability of the guided self-help CBT resource to patients, parents, and clinicians informing the design of a future RCT. If in a subsequent RCT the guided self-help CBT resource is found to be effective at reducing dental anxiety, these findings would be relevant to both users (young people and their parents) and service providers (dental practitioners and NHS).

One key issue to consider throughout this study is the involvement of children and young people themselves in the development, modification, and evaluation of the resource. Involving children throughout the research process is important as they have their own perspectives and these should be taken onto account whilst making decisions about their care [50]. A recent systematic review suggested the need for greater child-centred oral health research especially for evaluating the effectiveness of clinical interventions which could have a positive impact on the health outcomes for children and young people [51]. Additionally, engaging children in research enables age-appropriate measures to be chosen, ensures sustainable recruitment strategies, and assists in better dissemination [41, 52].

In terms of participant comfort, it is not anticipated that participants will feel distressed during the course of the study. However, discussing dental anxiety could lead a participant to consider a traumatic past dental experience and its impact. If a participant does become upset, the interview will be stopped until the participant feels able to continue. The researcher conducting the interviews will have the appropriate training and experience to support young people who have suffered unfavourable dental experiences. If through involvement in the study a participant is identified as having an undiagnosed psychological disorder, this will be dealt with by the clinical dental teams and a referral will be made to the Child Adolescent Mental Health Service.

Practical issues involved in performing the study will be challenges of involving general dental practices in research. These include ensuring early and continued involvement of clinic staff in the research study, developing the research capacity within the dental practice, e.g. training in research methodology, and maintaining enthusiasm for the research to ensure progress and momentum of the study [53].

The study findings will be widely disseminated through presentations at national and international dental and psychotherapy conferences and publications in peer-reviewed journals. A wider programme of dissemination will involve patients and the public. The findings will be disseminated back to participating young people and their parents in an easy read report. The findings will also be posted on the ‘dental fear in children’ thread of the Dental Fear Central forum, an international online resource for those with dental anxiety and dental professionals. A project steering group will be convened to include two members of the Sheffield Health watch and a parent representative as well as the research team and collaborators. The project steering group will meet every 6 months during the project. A panel of five young people (aged 11–16 years) will also be assembled and will meet twice during the project. The project steering group and young people’s panel will be involved in designing participant information leaflets, consent forms, questionnaires, and the topic guides for the qualitative interviews. The steering group and panel will also discuss and help interpret the results of the study, advise on dissemination, and make recommendations for the design of the pilot trial to evaluate the effectiveness of the resultant CBT resource.

## Abbreviations

CBT: cognitive behavioural therapy
NHS: National Health Service
RCT: randomised control trial
